# Detecting Adverse Drug Events Through the Chronological Relationship Between the Medication Period and the Presence of Adverse Reactions From Electronic Medical Record Systems: Observational Study

**DOI:** 10.2196/28763

**Published:** 2021-11-01

**Authors:** Kei Teramoto, Toshihiro Takeda, Naoki Mihara, Yoshie Shimai, Shirou Manabe, Shigeki Kuwata, Hiroshi Kondoh, Yasushi Matsumura

**Affiliations:** 1 Department of Medical Informatics Graduate School of Medicine Osaka University Suita Japan; 2 Division of Medical Informatics Tottori University Hospital Yonago Japan; 3 Department of Medical Informatics National Cancer Center Hospital Tokyo Japan; 4 Department of Clinical Information Management Nara City Hospital Nara Japan

**Keywords:** real world data, electronic medical record, adverse drug event

## Abstract

**Background:**

Medicines may cause various adverse reactions. An enormous amount of money and effort is spent investigating adverse drug events (ADEs) in clinical trials and postmarketing surveillance. Real-world data from multiple electronic medical records (EMRs) can make it easy to understand the ADEs that occur in actual patients.

**Objective:**

In this study, we generated a patient medication history database from physician orders recorded in EMRs, which allowed the period of medication to be clearly identified.

**Methods:**

We developed a method for detecting ADEs based on the chronological relationship between the presence of an adverse event and the medication period. To verify our method, we detected ADEs with alanine aminotransferase elevation in patients receiving aspirin, clopidogrel, and ticlopidine. The accuracy of the detection was evaluated with a chart review and by comparison with the Roussel Uclaf Causality Assessment Method (RUCAM), which is a standard method for detecting drug-induced liver injury.

**Results:**

The calculated rates of ADE with ALT elevation in patients receiving aspirin, clopidogrel, and ticlopidine were 3.33% (868/26,059 patients), 3.70% (188/5076 patients), and 5.69% (226/3974 patients), respectively, which were in line with the rates of previous reports. We reviewed the medical records of the patients in whom ADEs were detected. Our method accurately predicted ADEs in 90% (27/30patients) treated with aspirin, 100% (9/9 patients) treated with clopidogrel, and 100% (4/4 patients) treated with ticlopidine. Only 3 ADEs that were detected by the RUCAM were not detected by our method.

**Conclusions:**

These findings demonstrate that the present method is effective for detecting ADEs based on EMR data.

## Introduction

The investigation of adverse events in clinical trials and postmarketing surveillance requires an enormous amount of money and effort [1-3]. As clinical trials are performed with limited numbers of participants and limited investigation periods, they do not always clearly identify the full range of possible adverse events [4-6]. Although postmarketing surveillance, which is executed by specialized agencies in many countries, has focused on gathering information on adverse drug events (ADEs), the identification of ADEs in actual clinical settings remains insufficient due to its dependence upon voluntary reporting [7-11]. The introduction of electronic medical records (EMRs) by many hospitals has allowed for the secondary use of EMR data from multiple hospitals [12-15]. This enables a greater understanding of the ADEs that occur in actual patients without the costs associated with the traditional methods of determining the incidence of adverse events.

The occurrence of ADEs can be detected based on the chronological relationship between the presence of the adverse event and the medication period. The key data for the detection of an ADE are the date when a patient started to take the medicine and the date on which the medication was discontinued. It is not easy to accurately determine the medication period based on patient records because the medication data obtained from EMRs are based on a computer physician order entry (CPOE) system in which prescription orders are created for each prescription. In the clinical setting, physicians usually consider the amount of remaining medicine due to missed doses or overlapping previous prescriptions when they are preparing the prescription order. In the present study, we developed a medication history database in which both the start and end dates of medication were determined by combining the prescription order data according to the estimated amount of remaining medicine. To verify our ADE detection method, we focused on identifying ADEs with alanine aminotransferase (ALT) elevation using the medication history database and the serum ALT values obtained from the EMR. The accuracy of the detection of ADEs was examined by a review of medical records and by comparison with the Roussel Uclaf Causality Assessment Method (RUCAM), which is a standard method for detecting drug-induced liver injury (DILI) [16-25].

## Methods

### Experimental Environment

This study was performed in accordance with the World Medical Association Declaration of Helsinki, and the study protocol was approved by the institutional review board of the Osaka University Hospital (OUH), National Cerebral and Cardiovascular Center (NCVC), and Tottori University Hospital (TUH). This study was an observational study and did not obtain individual informed consent from the participants included in the study. However, the study protocol was posted on our webpage, giving the study participants an opportunity to opt out.

**Figure 1:**
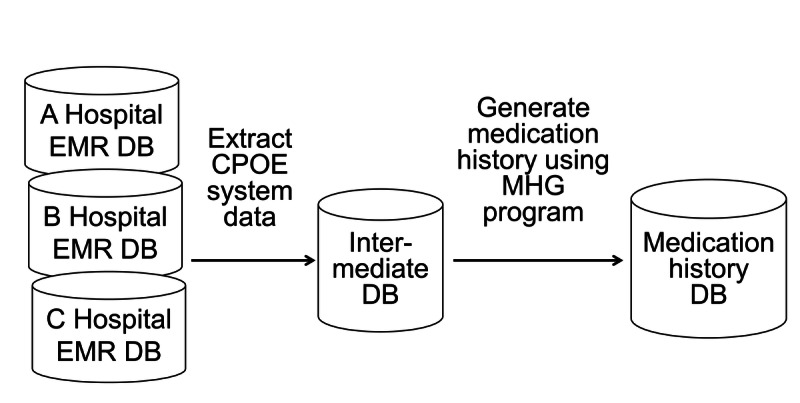
Procedure for generating the medication history. The data were extracted with an individually customized Structured Query Language from each CPOE database in the different medical facilities and transferred to an intermediate database. The MHG program was applied to the data in the intermediate database to generate the medication history. DB: database; CPOE: computer physician order entry; EMR: electronic medical record; MHG: medication history generation.

Because each CPOE system has its own database, the systems have different structures. We first developed an intermediate database to unify the database structure. The data from the original CPOE database were transferred to this intermediate database. We then generated the medication history by applying a medication history generation (MHG) program to the intermediate database (Figure 1). The medication history generation program was developed with Microsoft Visual Basic for Applications 7.0, and Microsoft Access 2010 was used for the intermediate database. Both the program and the database were installed on a laptop PC ( Intel Core i7-2640M CPU; 8 GB of memory) with the Microsoft Windows operating system.

### Generation of the Medication History

**Figure 2:**
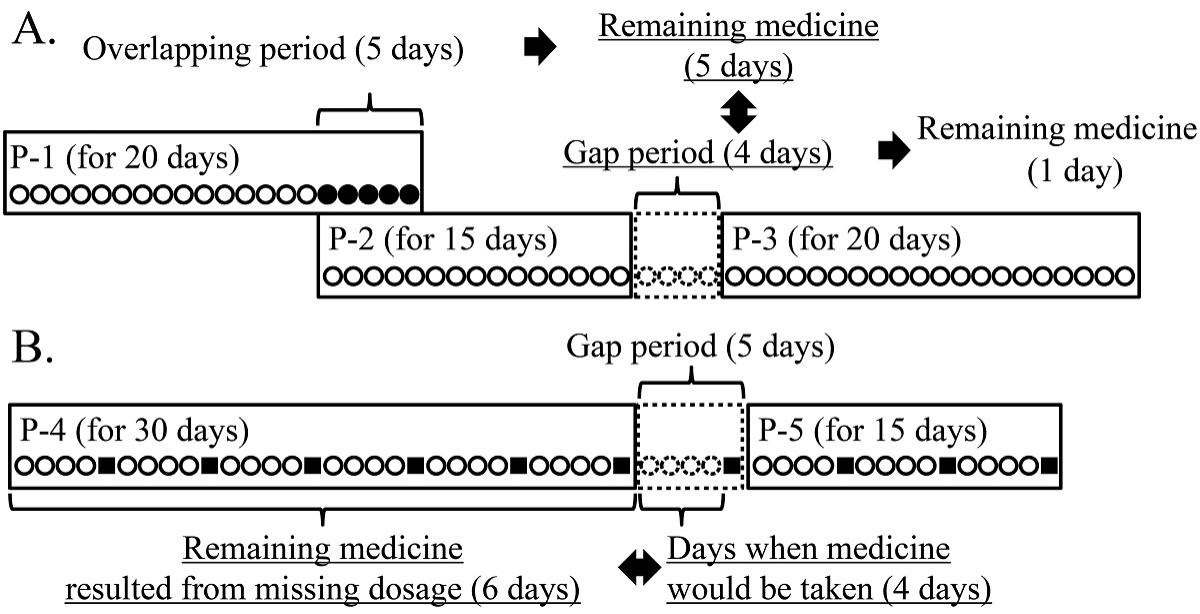
Process to generate a medication history. A. Generation of medication history with consideration to the overlapped period and gap period. The prescription order records (P-1, P-2, and P-3) were combined if the calculated remaining medicine was more than that needed for the days of the gap period. B. The medication history was generated under consideration of missing doses, assuming that missing doses occur once in 5 days. The prescription order records (P-4 and P-5) were combined if the amount of a remaining medicine was more than that needed for days of the gap period. Open circles indicate the days on which the patient took the medicine. Closed circles indicate the days in which the prescription orders overlapped. Closed squares indicate the days of missing doses.

The medication history includes the start and the end dates of medication for each medicine prescribed to a patient. To construct the medication history database, the CPOE records were combined with consideration to the remaining medicine (Figure 2).

First, the CPOE records for each medicine taken by an individual patient were extracted and combined sequentially from the oldest record to the newest record. As shown in Figure 2A, in cases where the last day of prescription 1 (P-1) was after the first day of P-2, the first day of P-1 was set as the start date of medication while the last day of P-2 was set as the end date, and the amount of the remaining medicine was estimated. In the case that a gap period lay between the last day of P-2 and the first day of P-3, if the amount of the remaining medicine was not less than the amount of medicine that would have been consumed during the gap period, P-2 and P-3 were combined.

We estimated the amount of the remaining medicines due to noncompliance by the patient, assuming that the rate of missed doses was constant. Accordingly, we set an unused medicine index (UMI), which indicated the rate of missing doses as a ratio of the period in which patients actually took the medicine to the prescription period. The amount of remaining medicine due to missing doses in P-4 was calculated (Figure 2B). If the amount of the remaining medicine was greater than that needed for the days during the gap period, P-4 and P-5 were combined. Figure 2 shows the algorithm for generating the medication history database.

The appropriate UMI value was determined by generating the medication history records for 9 medicines (pravastatin, cilostazol, isosorbide, nifedipine, ursodeoxycholic acid, rebamipide, amlodipine, aspirin, and methylcobalamin), which were considered to be long-term prescriptions. We set the UMI value as 1.4 because unnatural short-term gap periods tended to be observed when the UMI was <1.3 and because gap periods of a few months (considered to be the cessation of medicine) tended to be combined when the UMI was >1.5. To evaluate the validity of the UMI, we randomly selected 9 patients who had been treated for cardiovascular disease for >5 years and generated 725 medication history records. The medication history records were reviewed by the chief physicians, resulting in 98% of the records being considered appropriate.

### Detection of ADEs with Serum ALT Elevation

We detected the occurrence of ADEs based on the chronological relationship between the presence of the adverse event and the medication period. In this study, we focused on identifying ADEs with ALT elevation, which is known to reflect hepatocellular injury-type DILI. The elevation of ALT was selected because, in the RUCAM, the severity of hepatocellular injury-type DILI is defined by serum ALT. The ALT values were obtained from the laboratory test data in the EMR database. The criteria for the diagnosis of ADE with ALT elevation are shown in Table 1. ADEs with ALT elevation were detected during the medication period, and those with a decrease in the ALT level were detected after the cessation of the medication (criteria 1 and 2). If the elevated value decreased during the medication period, then the medicine was considered not to be causative; thus, it was excluded as a cause of ADE (criterion 3). Because it was difficult to distinguish an ALT elevation caused by a previous liver injury, viral hepatitis, or an operation from hepatocellular injury-type DILI, we excluded patients with any of these factors (criterion 4-III).

**Table 1:** Criteria for the diagnosis of hepatocellular injury with ALT elevation.

Criterion			Criterion details
**Inclusion criteria**			
	Elevation of ALT^a^ after initiation of medication	Peak ALT^b^> ULN^c^ of ALT and Peak ALT ≥ ALT (before start of medication)^d^ × 2	
	Decrease of ALT after cessation of medication	ALT (after cessation of medication)^e^ < Max ALT ^f^ × 0.5 or ALT (after cessation of medication) < ALT (ULN)	
**Exclusion criteria**			
	Decrease of ALT during the medication period but after the day of peak ALT	ALT (during medication period)^g^ < peak ALT × 0.25 or ALT (during medication period) < ULN of ALT	
	Liver injury induced by nondrug causes	Previous liver injury^h^, viral hepatitis^i^, or surgical operation^j^	

^a^ALT: alanine aminotransferase.

^b^Highest ALT value within 90 days from the start of medication.

^c^ULN: upper limit of normal.

^d^ALT value on the last day before the initiation of medication.

^e^Lowest ALT value within 30 days after the cessation of medication.

^f^Maximum ALT value during the medication period.

^g^Lowest ALT value during the medication period from the day of peak ALT to within 30 days from the date of medication cessation.

^h^Patients whose electronic medical records showed the following diseases (International Classification of Disease code 10): alcohol dependence (F10), liver disease (K70-K77), and gallbladder and bible duct disease (K80-K87).

^i^Patients whose electronic medical records showed positive results in the following laboratory blood tests: viral hepatitis A, B, and C ( immunoglobulin M antibody to hepatitis A virus antigen, hepatitis B surface antigen, hepatitis C virus core antigen); cytomegalovirus; and Epstein-Barr virus.

^j^Patients whose EMRs indicated that they had undergone surgery within 14 days before the day of peak ALT.

### The Study Population and the Target Medicines

In the present study, EMR data were obtained from 3 medical facilities: OUH, NCVC, and TUH. These medical institutions have independent EMR systems. In the study period, the data from a total of 1,587,939 patients were registered, and the total number of CPOE records was 37,935,783 (an average of 23.9 records per patient; Table 2).

**Table 2:** The medical facilities in the present study.

Characteristic	OUH^a^	NCVC^b^	TUH^c^
Manufacturer	NEC Corp.	NEC Corp.	IBM Corp.
CPOE^d^ database model	Oracle	Oracle	Database 2
Data range (mm/dd/yy)	04/01/00-12/01/12	04/01/00-02/01/14	0/01/03-09/01/13
Patients, n	1,028,852	251,143	307,944
CPOE records, n	20,447,443	8,128,059	9,360,281

^a^OUH: Osaka University Hospital.

^b^NCVC: National Cerebral and Cardiovascular Center.

^c^TUH: Tottori University Hospital.

^d^CPOE; computer physician order entry.

The target medicines were aspirin, clopidogrel, and ticlopidine. These are antiplatelet drugs that have been reported to cause hepatocellular injury-type DILI [26-28]. Earlier studies have suggested that clopidogrel is associated with a lower risk of hepatocellular injury-type DILI in comparison to ticlopidine [29].

### The Rates of ADE With ALT Elevation With Each Target Medicine

To calculate the rates of ADE with ALT elevation that occurred with each medicine, we counted the number of patients who met the diagnostic criteria (Table 1). The severity of ADE with ALT elevation was categorized according to the maximum ALT value as mild elevation (maximum ALT ≥40 IU/L), moderate elevation (maximum ALT ≥80 IU/L), and severe elevation (maximum ALT ≥200 IU/L). The rate of ADEs with ALT elevation was calculated by dividing the number of ADE patients by the number of patients who took the targeted medicine, and the ALT values were tested at least 3 times (before, during, and after the medication period).

### Evaluating Results That Were Indicative of ADE With ALT Elevation.

We selected the patients with moderate and severe ALT elevation (maximum ALT ≥80 IU/L) whose medical records were recorded electronically at OUH and TUH and checked the progress notes recorded from 3 days before to 3 days after the date of the peak ALT value. The numbers of medical records subjected to review for each of the drugs were as follows: aspirin (n=83), clopidogrel (n=29), and ticlopidine (n=8). These records were used to determine whether or not the elevation of ALT was due to an ADE. The ADE cases were categorized into 3 groups: (1) ADE caused by the targeted medicine, (2) ADE caused by a concomitant medicine, and (3) offending medicine not identified.

### Comparison of the Detection of ADE With ALT Elevation Between Our Proposed Method and the RUCAM

The RUCAM is the standard method for detecting DILI. The RUCAM uses a 5-stage scoring system to assess the possibility of DILI by classifying the condition as hepatocellular, cholestatic, or mixed based on the laboratory test data and clinical data.

We compared the accuracy of detecting hepatocellular-type ADE between our method and the RUCAM. Patients with ALT levels of >200 were included in the analysis (10,608 patients from OUH and 5464 patients from TUH).

The primary screening was performed to select hepatocellular-type ADE for the RUCAM. The screening criterion was as follows: ALT level >200 and (ALT/upper limit of normal/(alkaline phosphatase/upper limit of normal)>5 within 90 days of the first day of using the verified medication. Next, we determined the RUCAM score based on a review of medical records. Probable and highly probable scores according to the RUCAM system were classified as hepatocellular-type DILI in this study.

### Statistical Analysis

Multiple comparisons were performed using the Ryan method, and the Fisher exact test was used to compare the rates of ADE. *P* values of <0.05 were considered to indicate statistical significance*.* All statistical analyses were performed using the R software version 3.1.2 (The R Foundation for Statistical Computing).

## Results

Table 3 shows a summary of the medication history records for the target medicines that were generated by our system. Aspirin was the most frequently used medication in our study population. The numbers of patients who were treated with clopidogrel and ticlopidine were approximately equal. The CPOE records were combined into a single medication history record in 8.80% (58,873/668,765), 13.81% (12,224/88,520 patients), and 8.51% (8654/104,003) of the patients treated with aspirin, clopidogrel, and ticlopidine, respectively, which indicated that the medication histories were correctly generated.

**Table 3:** The medication histories generated for the target medicines (N=1,587,939).

Values	Aspirin	Clopidogrel	Ticlopidine
Patients, n (%^a^)	40,938 (2.58)	10,263 (0.65)	6224 (0.39)
CPOE^b^ records, n	668,765	88,520	104,003
CPOE records per patient, mean	16.3	8.6	16.7
Medication history records, n	58,873	12,224	8,854
Medication history records per patient, mean	1.4	1.2	1.4

^a^Percentage of the study population treated with the target medicine/electronic medical record–registered population (1,5879,939 patients).

^b^CPOE: computer physician order entry.

The rate of ADEs with ALT elevation among patients who received ticlopidine was significantly higher than that among patients who received the other 2 medicines (Table 4). The rates of ADE with ALT elevation in patients who received aspirin and clopidogrel did not differ to a statistically significant extent. The rates of severe ALT elevation with each of the target medicines showed the same tendency.

We reviewed the medical records of the patients in whom an ADE with ALT elevation was detected by our system (Table 5). The number of records subjected to review for each of the drugs was 83 for aspirin, 29 for clopidogrel, and 8 for ticlopidine. The number of records in which the cause of liver injury was described was 30 for aspirin, 9 for clopidogrel, and 4 for ticlopidine. Among these, the number of records in which an ADE with ALT elevation was diagnosed was 27 (90%) for aspirin, 9 (100%) for clopidogrel, and 4 (100%) for ticlopidine. These findings demonstrated that the method of the present study was appropriate for detecting ADE with ALT elevation. However, the causative medicines of ADEs with ALT elevation described in the medical records were not only the target medicine but also concomitant medicines. There were cases in which the offending medicine was not specified. In the cases in which the concomitant medicine was described as the causative medicine of an ADE with ALT elevation, the target medicine was also thought to be a candidate based on the chronological pattern of the medication period and ALT elevation. This may be due to physicians suspecting an ADE and then discontinuing all of the possible causative medicines.

**Table 4:** The rates of adverse drug events with ALT elevation.

Patients		Aspirin		Clopidogrel		Ticlopidine			
Target patient distribution, n		26,059		5076		3974			
**DILI^a^ patients**									
	(MAX^b^ ALT^c^ >ULN^c^)		868 (3.33%)		188 (3.70%)		226 (5.69%)^e^		
	MAX ALT ≥ 80 IU/L		341 (0.95%)		69 (0.93%)		83 (1.43%)^e^		
	MAX ALT ≥ 200 IU/L		93 (0.36%)		22 (0.43%)		26 (0.65%)^f^		

^a^DILI: drug-induced liver injury.

^b^MAX: maximum.

^c^ALT: alanine aminotransferase.

^d^ULN: upper limit of normal.

^e^*P*<.001 vs other groups.

^f^*P*<.001 vs Aspirin.

**Table 5:** Evaluation by review of medical records.

Medical record values			Aspirin		Clopidogrel		Ticlopidine
**ADEs^a^ with ALT^b^ elevation, n**			27		9		4
	Caused by target medicine	8		6		1	
	Caused by concomitant medicine	11		1		2	
	Offending medicine not specified	8		2		1	
Other causes of liver injury, n			3		0		0
Total, n			30		9		4

^a^ADE: adverse drug event.

^b^ALT: alanine aminotransferase.

The number of patients diagnosed with hepatocellular-type ADE with our proposed method and the RUCAM are shown in Table 6. The first RUCAM screening identified 10 patients at OUH and 39 patients at TUH as candidates of hepatocellular-type ADE. The number of candidate patients was very few at OUH because the testing rate of alkaline phosphatase (ALP) within 90 days from starting the medication was very low (882/16,735, 5.26%) for OUH. As a result, none of the patients were suspected as hepatocellular-type ADE at OUH, while 51 patients were suspected as hepatocellular-type ADE by our method. On the other hand, the rate of ALP testing within 90 days from starting the medication was not low at TUH (6692/9097, 73.56%). At TUH, 11 patients were detected as DILI by both our method and the RUCAM. Two patients were detected as hepatocellular-type ADE only by our method, and both patients were thought to be hepatocellular-type ADE by the review of medical records. Three patients were not detected as hepatocellular-type ADE by our method because the ALT levels of these patients did not recover within 30 days of termination of the medication (within 33 days, 40 days, and 45 days, respectively).

**Table 6:** ADE with alanine aminotransferase level elevation detection results by RUCAM and the proposed method.

Values			Aspirin					Clopidogrel					Ticlopidine					Total		
			OUH^a^		TUH^b^		OUH			TUH		OUH			TUH		OUH			TUH
Target patients			7611		4002		1266			951		1731			511		10,608			5464
**RUCAM^c^**																				
	First^d^ screening	5		28		3			9		2			2		10			39	
	ADE^e,f^	0		10		0			3		0			1		0			14	
ADE medication history^g^			26		10		7			2		18			1		51			13

^a^OUH: Osaka University Hospital.

^b^TUH: Tottori University Hospital.

^c^RUCAM: Roussel Uclaf Causality Assessment Method.

^d^Alanine aminotransferase level >200 and (alkaline phosphatase /200)/(alanine aminotransferase/40) <5.

^e^ADE: adverse drug event.

^f^The number of patients diagnosed with “probable” suspected of drug-induced liver injury or with a degree greater than “probable” by the RUCAM (alanine aminotransferase ALT level >200) includes first screening patients.

^g^The number of patients diagnosed with an ADE by the proposed method (alanine aminotransferase level >200).

## Discussion

### Principal Findings

Accurate demonstration of the start and end dates of a medication period is important in pharmacoepidemiologic research. However, the CPOE records in EMRs cannot clearly demonstrate the total duration of the medication period. In the present study, we generated a medication history database from the CPOE databases of 3 hospitals and systematically diagnosed ADEs with ALT elevation according to the chronological relationship between the changes in ALT values and the duration of medication using a medication history database. Because the medication history database can be applied not only to the detection of ADEs but also to crossover studies that compare drug efficacy in the same patients, it can become a basis for pharmacoepidemiologic research.

The comparison of the RUCAM and our method revealed that the rates of ALT and ALP testing influenced the accuracy of the RUCAM in the detection of ADEs. In a prospective study, laboratory test data can be obtained according to a research plan. However, in a retrospective study, missing data often become problematic. Scoring in the RUCAM requires information such as the use of concomitant medications, drug risk information, the presence or absence of a rechallenge, and the history of alcohol consumption. This information is not registered as structured data in EMRs. In this study, a review of medical records was needed to determine the score for the RUCAM. In contrast, our method used only standardized data, such as laboratory test data, prescription data, disease name data, and surgical data. For this reason, our method is applicable to the detection of ADEs in a retrospective analysis of big data generated by EMRs.

The population characteristics greatly affect the rate of adverse events. In clinical trials, the incidence of adverse events may be accurate because blood testing is routinely performed in all patients. On the other hand, in observational studies, the timing of blood testing differs for each patient. There may be great differences in the rates of adverse events depending on how the study population is defined. A previous clinical study in Japan reported that the rates of serious liver injury among patients receiving ticlopidine and clopidogrel were 13.6% (129/948) and 5.1% (115/2261), respectively [30,31]. However, these studies had different study populations, and caution must be exercised when interpreting the comparison of the rates of adverse events. The present method determined the rates of adverse events for some medicines under the same conditions for ticlopidine (188/5076 ,3.70%) and clopidogrel (226/3974, 5.69%); thus, this method could be used to compare the risk of adverse events between medicines (ticlopidine therapy is associated with a greater risk of developing ADEs in comparison to clopidogrel).

When physicians suspect an ADE with ALT elevation, all of the medicines that might have caused the ADE are likely to be discontinued. Thus, it was difficult to differentiate the causative medicine from the concomitant medicines using our method. Our method demonstrated the maximum rate of ADEs with ALT elevation induced by a targeted medicine, assuming that the targeted medicine was the causative medicine in all cases.

Although aspirin has been reported as a cause of liver injury, the rate in Asian populations remains unclear. According to the clopidogrel versus aspirin in patients at risk of ischemic events (CAPRIE) Steering Committee report, the rates of liver injury in patients receiving aspirin and clopidogrel were 2.97% (285/9599) and 3.15% (302/9586), respectively, which are in line with the rates obtained in the present study (aspirin: 868/26,059, 3.33%; clopidogre: 188/5076, 3.70%) [32]. The rates of severe liver injury in the same report were 0.19% for aspirin (93/5076, 0.36% in this study) and 0.11% for clopidogrel (22/5076, 0.43% in this study). Similar to our study, the rates of severe liver injury did not differ between patients using aspirin and those using clopidogrel.

Even though the absolute risk of a medicine is difficult to estimate, our method can estimate the upper limit of the risk. Furthermore, for some medicines, our method can estimate the risk of for ADE with ALT elevation one at a time under the same conditions, and the risk can be compared among different medicines.

### Limitations

In this study, we used the medication history database created from CPOE records to detect DILI, but we did not detect all cases of DILI. First, we focused on elevated serum ALT levels. Elevated serum ALT can capture hepatocellular-type DILI, but it may not detect cholestatic-type DILI, which is characterized by elevation of the serum ALP level. Second, we were not able to detect DILI that did not meet our diagnostic criteria, such as delayed DILI, even the hepatocellular-type DILI. This type of detection requires a different set of criteria.

### Conclusions

The generation of a medication history database enabled us to detect ADEs with ALT elevation through the chronological relationship between the medication period and occurrence of liver injury. As our method used only standardized data from EMRs, it was possible to analyze real-world data accumulated by EMRs in multiple hospitals. Although our method could not identify the causative medicine among concomitant medicines, it was possible to compare the risk of ADEs for different medicines.

## Abbreviations

ADEs: adverse drug events
ALP: alkaline phosphatase
ALT: alanine aminotransferase
CAPRIE: clopidogrel versus aspirin in patients at risk of ischemic events
CPOE: computer physician order entry
DILI: drug-induced liver injury
EMR: electronic medical record
MHG: medication history generation
NCVC: National Cerebral and Cardiovascular Center
OUH: Osaka University Hospital
RUCAM: Roussel Uclaf Causality Assessment Method
TUH: Tottori University Hospital
UMI: unused medicine index

## References

[1] Vouk K, Benter U, Amonkar MM, Marocco A, Stapelkamp C, Pfersch S, Benjamin L (2016). Cost and economic burden of adverse events associated with metastatic melanoma treatments in five countries. J Med Econ.

[2] Stark RG, John J, Leidl R (2011). Health care use and costs of adverse drug events emerging from outpatient treatment in Germany: a modelling approach. BMC Health Serv Res.

[3] Arondekar B, Curkendall S, Monberg M, Mirakhur B, Oglesby AK, Lenhart GM, Meyer N (2015). Economic burden associated with adverse events in patients with metastatic melanoma. J Manag Care Spec Pharm.

[4] Lasser KE, Allen PD, Woolhandler SJ, Himmelstein DU, Wolfe SM, Bor DH (2002). Timing of new black box warnings and withdrawals for prescription medications. JAMA.

[5] Jefferys DB, Leakey D, Lewis JA, Payne S, Rawlins MD (1998). New active substances authorized in the United Kingdom between 1972 and 1994. Br J Clin Pharmacol.

[6] Frank C, Himmelstein DU, Woolhandler S, Bor DH, Wolfe SM, Heymann O, Zallman L, Lasser KE (2014). Era of faster FDA drug approval has also seen increased black-box warnings and market withdrawals. Health Aff (Millwood).

[7] Wysowski DK, Swartz L (2005). Adverse drug event surveillance and drug withdrawals in the United States, 1969-2002: the importance of reporting suspected reactions. Arch Intern Med.

[8] Simone LK, Brumbaugh J, Ricketts C (2014). Medical devices, the FDA, and the home healthcare clinician. Home Healthc Nurse.

[9] Hojo T (2017). Regulatory science in practice (Pharmaceuticals and Medical Devices Agency). Yakugaku Zasshi.

[10] Moore TJ, Furberg CD, Mattison DR, Cohen MR (2016). Completeness of serious adverse drug event reports received by the US Food and Drug Administration in 2014. Pharmacoepidemiol Drug Saf.

[11] Hazell L, Shakir SAW (2006). Under-reporting of adverse drug reactions : a systematic review. Drug Saf.

[12] Shin J, Hunt CM, Suzuki A, Papay JI, Beach KJ, Cheetham TC (2013). Characterizing phenotypes and outcomes of drug-associated liver injury using electronic medical record data. Pharmacoepidemiol Drug Saf.

[13] Dandala B, Joopudi V, Tsou C, Liang JJ, Suryanarayanan P (2020). Extraction of information related to drug safety surveillance from electronic health record notes: joint modeling of entities and relations using knowledge-aware neural attentive models. JMIR Med Inform.

[14] Munkhdalai T, Liu F, Yu H (2018). Clinical relation extraction toward drug safety surveillance using electronic health record narratives: classical learning versus deep learning. JMIR Public Health Surveill.

[15] Ujiie Shogo, Yada Shuntaro, Wakamiya Shoko, Aramaki Eiji (2020). Identification of adverse drug event-related Japanese articles: natural language processing analysis. JMIR Med Inform.

[16] Onakpoya IJ, Heneghan CJ, Aronson JK (2016). Post-marketing withdrawal of 462 medicinal products because of adverse drug reactions: a systematic review of the world literature. BMC Med.

[17] Hassan A, Fontana RJ (2019). The diagnosis and management of idiosyncratic drug-induced liver injury. Liver Int.

[18] Leise MD, Poterucha JJ, Talwalkar JA (2014). Drug-induced liver injury. Mayo Clin Proc.

[19] Nathwani RA, Pais S, Reynolds TB, Kaplowitz N (2005). Serum alanine aminotransferase in skeletal muscle diseases. Hepatology.

[20] Green RM, Flamm S (2002). AGA technical review on the evaluation of liver chemistry tests. Gastroenterology.

[21] Rockey DC, Seeff LB, Rochon J, Freston J, Chalasani N, Bonacini M, Fontana RJ, Hayashi PH, US Drug-Induced Liver Injury Network (2010). Causality assessment in drug-induced liver injury using a structured expert opinion process: comparison to the Roussel-Uclaf causality assessment method. Hepatology.

[22] Rochon J, Protiva P, Seeff LB, Fontana RJ, Liangpunsakul S, Watkins PB, Davern T, McHutchison JG, Drug-Induced Liver Injury Network (DILIN) (2008). Reliability of the Roussel Uclaf Causality Assessment Method for assessing causality in drug-induced liver injury. Hepatology.

[23] Chalasani NP, Hayashi PH, Bonkovsky HL, Navarro VJ, Lee WM, Fontana RJ, Practice Parameters Committee of the American College of Gastroenterology (2014). ACG Clinical Guideline: the diagnosis and management of idiosyncratic drug-induced liver injury. Am J Gastroenterol.

[24] Benichou C, Danan G, Flahault A (1993). Causality assessment of adverse reactions to drugs—II. An original model for validation of drug causality assessment methods: case reports with positive rechallenge. J Clin Epidemiol.

[25] Cheetham TC, Lee J, Hunt CM, Niu F, Reisinger S, Murray R, Powell G, Papay J (2014). An automated causality assessment algorithm to detect drug-induced liver injury in electronic medical record data. Pharmacoepidemiol Drug Saf.

[26] Pisapia R, Abdeddaim A, Mariano A, Rianda A, Vincenzi L, Taibi C, Baiocchini A, Del Nonno F, DʼOffizi G (2015). Acute hepatitis associated with clopidogrel: a case report and review of the literature. Am J Ther.

[27] Motola D, Biagi C, Leone R, Venegoni M, Lapi F, Cutroneo P, Vargiu A, Bonaiuti R, Montanaro N, Vaccheri A (2012). Ticlopidine safety profile: a case/non-case study on the basis of the spontaneous ADRs reporting in Italy. Curr Drug Saf.

[28] Laster J, Satoskar R (2014). Aspirin-induced acute liver injury. ACG Case Rep J.

[29] Shigematsu H, Komori K, Tanemoto K, Harada Y, Nakamura M (2012). Clopidogrel for Atherothrombotic Event Management in Patients with Peripheral Arterial Disease (COOPER) study: safety and efficacy of clopidogrel versus ticlopidine in Japanese patients. Ann Vasc Dis.

[30] Danan G, Benichou C (1993). Causality assessment of adverse reactions to drugs—I. A novel method based on the conclusions of international consensus meetings: application to drug-induced liver injuries. J Clin Epidemiol.

[31] K. Sanofi K.

[32] CAPRIE Steering Committee (1996). A randomised, blinded, trial of clopidogrel versus aspirin in patients at risk of ischaemic events (CAPRIE). CAPRIE Steering Committee. Lancet.

